# Supplementary material for: Apple Root Microbiome as Indicator of Plant Adaptation to Apple Replant Diseased Soils
Source: Microorganisms. 2023 May 24;11(6):1372. doi: 10.3390/microorganisms11061372 (PMC10301482; doi:10.3390/microorganisms11061372)
Supplement: Supplementary file 1 [file microorganisms-11-01372-s001.zip › sup figures.pptx]

## Slide 1
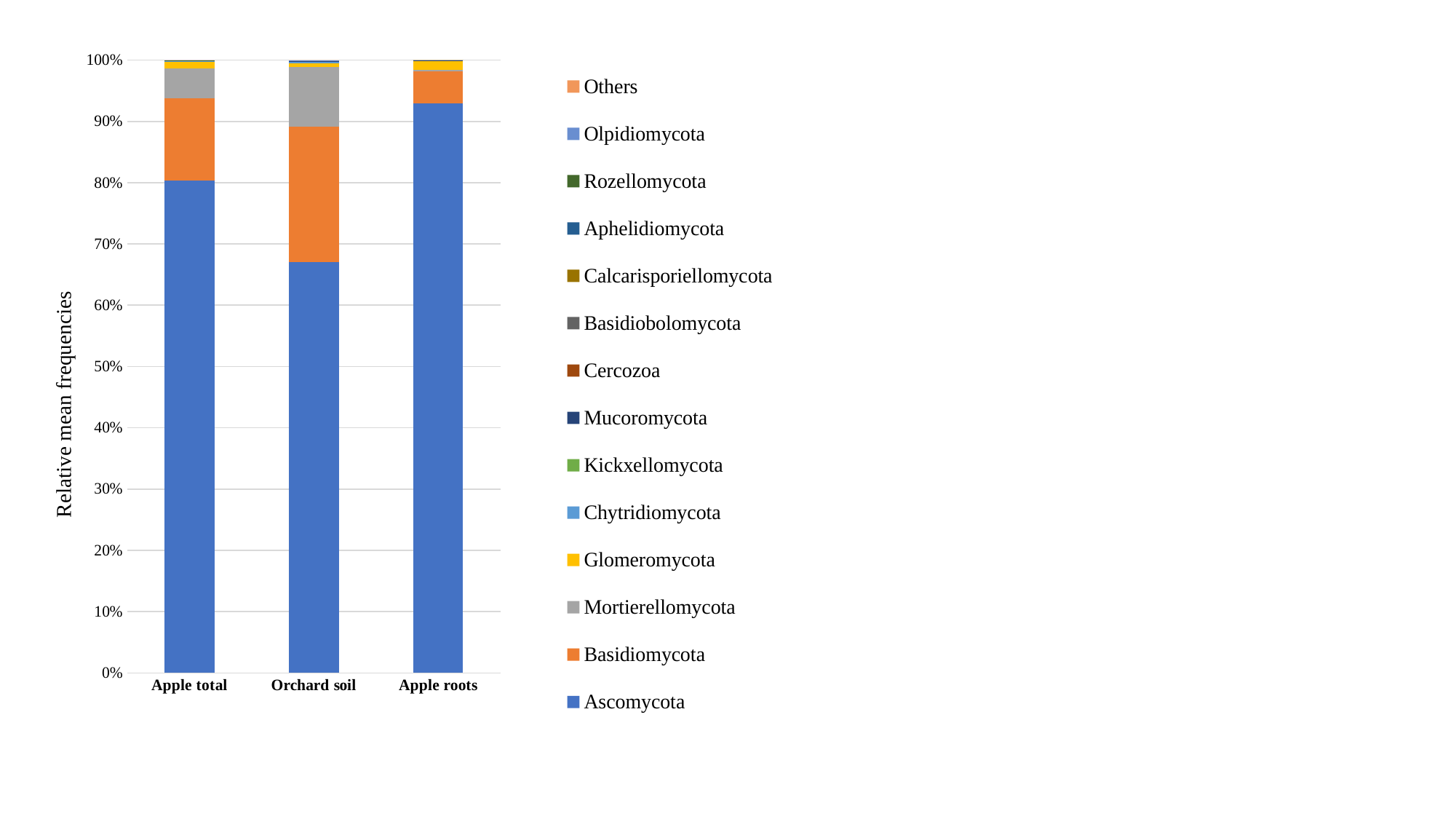

### Chart
| Category | Ascomycota | Basidiomycota | Mortierellomycota | Glomeromycota | Chytridiomycota | Kickxellomycota | Mucoromycota | Cercozoa | Basidiobolomycota | Calcarisporiellomycota | Aphelidiomycota | Rozellomycota | Olpidiomycota | Others |
|---|---|---|---|---|---|---|---|---|---|---|---|---|---|---|
| Apple total | 0.803659845023 | 0.134230081462 | 0.0482952513411 | 0.0111265646732 | 0.00122392211405 | 0.000466918338963 | 0.000353665805682 | 0.000337770713292 | 9.53705543413e-05 | 6.35803695609e-05 | 5.3645936817e-05 | 4.37115040731e-05 | 2.38426385853e-05 | 2.5829525335474557e-05 |
| Orchard soil | 0.670453243884 | 0.220657776323 | 0.0979505849628 | 0.00557146363413 | 0.00247484251002 | 0.000944939867463 | 0.000683138345742 | 0.000638141209196 | 0.000196351141291 | 0.000130900760861 | 0.000110447516976 | 8.59036243148e-05 | 4.90877853228e-05 | 5.317843488361618e-05 |
| Apple roots | 0.929466079431 | 0.0526039252048 | 0.00139854736517 | 0.0163730489878 | 4.24972956266e-05 | 1.54535620461e-05 | 4.24972956266e-05 | 5.40874671612e-05 | 0.0 | 0.0 | 0.0 | 3.86339051151e-06 | 0.0 | 2.581268532253489e-13 |Relative mean frequencies

## Slide 2
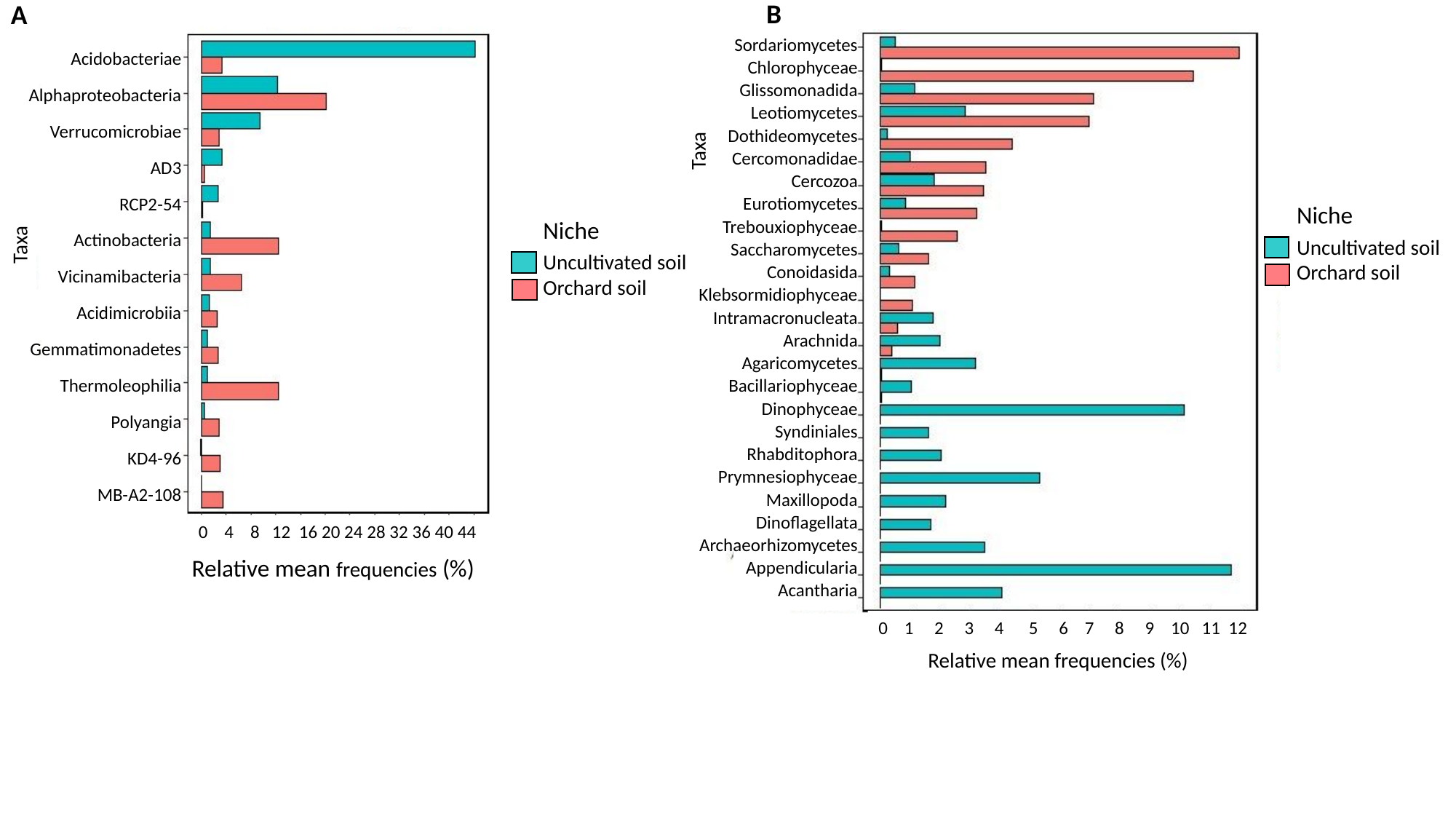

B
A
Sordariomycetes
Chlorophyceae
Glissomonadida
Leotiomycetes
Dothideomycetes
Cercomonadidae
Cercozoa
Eurotiomycetes
Trebouxiophyceae
Saccharomycetes
Conoidasida
Klebsormidiophyceae
Intramacronucleata
Arachnida
Agaricomycetes
Bacillariophyceae
Dinophyceae
Syndiniales
Rhabditophora
Prymnesiophyceae
Maxillopoda
Dinoflagellata
Archaeorhizomycetes
Appendicularia
Acantharia
Acidobacteriae
Alphaproteobacteria
Verrucomicrobiae
AD3
RCP2-54
Actinobacteria
Vicinamibacteria
Acidimicrobiia
Gemmatimonadetes
Thermoleophilia
Polyangia
KD4-96
MB-A2-108
 Taxa
Niche
Taxa
Niche
Uncultivated soil
Orchard soil
Uncultivated soil
Orchard soil
0 4 8 12 16 20 24 28 32 36 40 44
Relative mean frequencies (%)
0 1 2 3 4 5 6 7 8 9 10 11 12
Relative mean frequencies (%)

## Slide 3
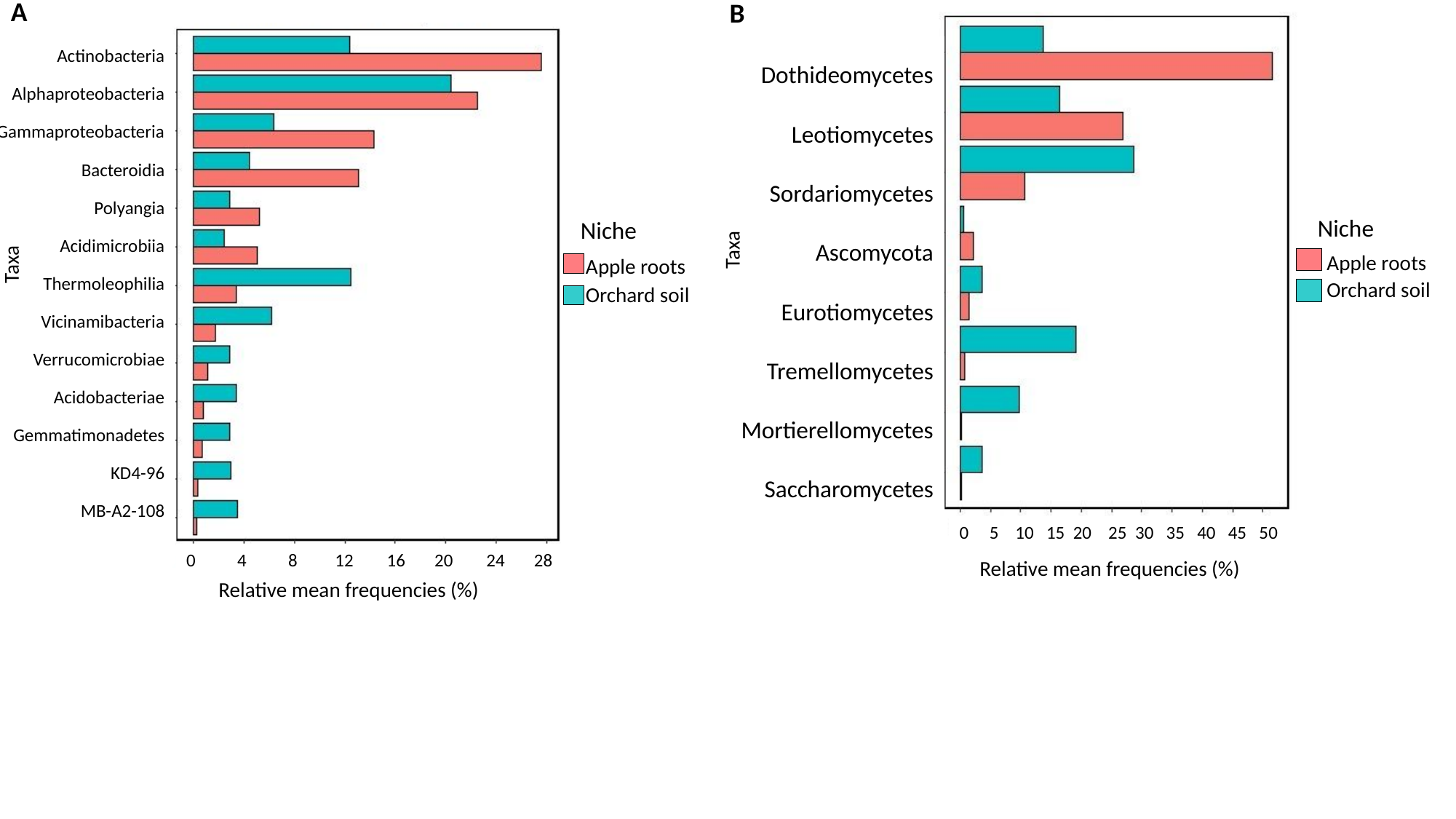

A
B
Actinobacteria
Alphaproteobacteria
Gammaproteobacteria
Bacteroidia
Polyangia
Acidimicrobiia
Thermoleophilia
Vicinamibacteria
Verrucomicrobiae
Acidobacteriae
Gemmatimonadetes
KD4-96
MB-A2-108
Dothideomycetes
Leotiomycetes
Sordariomycetes
Ascomycota
Eurotiomycetes
Tremellomycetes
Mortierellomycetes
Saccharomycetes
Taxa
Taxa
Niche
Niche
Taxa
Apple roots
Orchard soil
Apple roots
Orchard soil
Niche
Apple roots
Orchard soil
0 5 10 15 20 25 30 35 40 45 50
0 4 8 12 16 20 24 28
Relative mean frequencies (%)
Relative mean frequencies (%)
