# Supplementary material for: Apple Root Microbiome as Indicator of Plant Adaptation to Apple Replant Diseased Soils
Source: Microorganisms. 2023 May 24;11(6):1372. doi: 10.3390/microorganisms11061372 (PMC10301482; doi:10.3390/microorganisms11061372)
Supplement: Supplementary file 1 [file microorganisms-11-01372-s001.zip › Supplementary tables S1_S7.pdf]

Table S1. Data description for 16S, 18S and ITS2 analysis

**Amplicons**		**16S rRNA**	**18S rRNA**	**ITS2**
Total number of samples		81	53	72
After quality control and filtering	Number of reads	1,744,577	308,956	8,003,297
	Number of features	7,361	3,308	1,050
	Mean frequency	21,538	5,829	111,157
	Median frequency	20,009	5,302	88,185
After normalization	Rarefaction depth	5,900	1,434	7,190
	Number of samples	81	53	70
	Number of reads	477,900	76,002	503,300
	Number of features	7,360	3,149	1,050

Table S7. Orchard soil Shannon diversity

Amplicon/Location	16S rRNA	18S rRNA	ITS
Orchard_1	$9.0^{C} \pm 0.19$	$6.58^{AB} \pm 0.63$	$5.9^{AB} \pm 0.36$
Orchard_2	$9.3^{B} \pm 0.09$	$6.98^{A} \pm 0.44$	$6.2^{A} \pm 0.19$
Orchard_3	$9.5^{AB} \pm 0.09$	$6.8^{A} \pm 0.31$	$6.4^{A} \pm 0.23$
Orchard_4	$9.4^{AB} \pm 0.17$	$5.58^{AB} \pm 1.14$	$5.8^{AB} \pm 0.21$
Orchard_5	$9.4^{AB} \pm 0.12$	$5.18^{B} \pm 1.43$	$5.4^{B} \pm 0.62$
Orchard_6	$9.7^{A} \pm 0.14$	$5.88^{AB} \pm 0.75$	$6.2^{A} \pm 0.37$

For each variable, data followed different letter are significantly different according to Tukey's test ($P<0.05$)
